# MUC5AC and inflammatory mediators associated with respiratory outcomes in the British 1946 birth cohort

**DOI:** 10.1111/resp.12092

**Published:** 2013-07-25

**Authors:** Lauren Johnson, Imran Shah, Andrew X Loh, Lynne E Vinall, Ana S Teixeira, Karine Rousseau, John W Holloway, Rebecca Hardy, Dallas M Swallow

**Affiliations:** 1 Research Department of Genetics, Evolution and Environment, University College London Darwin BuildingLondon, UK; 2 MRC Unit for Lifelong Health & AgeingLondon, UK; 3 Faculty of Medicine, University of Southampton, Southampton General HospitalSouthampton, Hampshire, UK

**Keywords:** airway epithelium, asthma, genetics, inflammation, respiratory function test

## Abstract

***Background and objective:*** Dysregulation of respiratory mucins, MUC5AC in particular, has been implicated in respiratory disease and *MUC5AC* expression is up-regulated in response to environmental challenges and inflammatory mediators. The aim of this study was to examine the effect of genetic variation on susceptibility to common respiratory conditions.

***Methods:*** The association of *MUC5AC* and the closely linked genes *MUC2* and *MUC5B* with respiratory outcomes was tested in the MRC National Survey of Health and Development, a longitudinal birth cohort of men and women born in 1946. Also examined were the functional variants of the genes encoding inflammatory mediators, *IL13, IL1B, IL1RN, TNFA* and *ERBB1*, for which there is a likely influence on *MUC5AC* expression and were explored potential gene-gene interactions with these inflammatory mediators.

***Results:*** Statistically significant associations between the 3'ter *MUC5AC* simple nucleotide polymorphism (SNP) rs1132440 and various non-independent respiratory outcomes (bronchitis, wheeze, asthma, hay fever) were reported while the adjacent loci show slight (but largely non-statistically significant) differences, presumably reflective of linkage disequilibrium (allelic association) across the region. A novel association between bronchitis and a non-synonymous functional *ERBB1* SNP, rs2227983 (aka epidermal growth factor receptor:R497K, R521K) is also reported and evidence presented of interaction between *MUC5AC* and *ERBB1* and between *MUC5AC* and *IL1RN* with respect to bronchitis. The *ERBB1* result suggests a clear mechanism for a biological interaction in which the allelic variants of epidermal growth factor receptor differentially affect mucin expression.

***Conclusions:*** The *MUC5AC* association and the interactions with inflammatory mediators suggest that genetically determined differences in *MUC5AC* expression alter susceptibility to respiratory disease.

**SUMMARY AT A GLANCE**

This longitudinal cohort study shows occurrence of the common respiratory conditions bronchitis, wheeze, asthma and hay fever to be associated with genetic variation in a mucin gene, *MUC5AC*. Functional variation in the epidermal growth factor receptor (epidermal growth factor receptor encoded by *ERBB1*) is also associated with bronchitis and modulates the *MUC5AC* effect.

## Introduction

Disturbances to the normal dynamics of the respiratory epithelial layer as a result of allergens, microorganisms and noxious agents that cause inflammation often leads to secretion of large quantities of airway mucus which allows the expulsion of the offending agent. In chronic airway disease, this mechanism is intensely active for lengthy periods, exacerbating disease symptoms, and in severe asthma, some airways become irreversibly occluded.^1^ Genetic variation affecting any part of these pathways is likely to influence susceptibility and severity of respiratory symptoms.

The major high molecular-weight glycoprotein components of respiratory mucus are the mucins MUC5AC and MUC5B.^2^ The quantities of MUC5AC and MUC5B are increased in a number of respiratory conditions and experimental models^3^–^7^ and expression of their genes, *MUC5AC* and *MUC5B* are up-regulated.^6^,^8^

Several mediators of inflammation such as the cytokines, interleukin 13 (encoded by the gene *IL13*), interleukin 1B (encoded by *IL1B*) and tumour necrosis factor α (encoded by *TNFA*), which are present at high levels within the asthmatic airways,^9^–^13^ are thought to orchestrate respiratory mucus hypersecretion and are known to up-regulate *MUC5AC* expression as well as secretion,^14^–^17^ and *MUC5AC* is regulated through an epidermal growth factor receptor (EGFR, encoded by the gene known as *ERBB1*) signalling pathway.^7^,^8^,^18^–^22^ See Table S1 in the supporting information available online for details of functional evidence.

Previous studies showed that the genetically variable *MUC2* tandem repeat (TR) sequence of the main mucin domain had a different size distribution in atopic individuals with and without asthma.^23^ Although *MUC2* expression shows some evidence of up-regulation in inflammatory disease,^23^ biologically, *MUC2* appears a rather unlikely candidate for altering respiratory disease susceptibility because the protein is found at only very low levels in the airways.^24^ – ^25^ *MUC5AC* is located adjacent to *MUC2* in a region of strong linkage disequilibrium (allelic association) on chromosome 11p15.5.^26^ Thus the association seen between *MUC2* and asthma could in fact be a consequence of association between the *MUC2* TR and a causative allele (variant) in *MUC5AC*, or possibly *MUC5B*.

Here we explore the possible association between variants in these *MUC* genes (see Table S2 in the supporting information available online) and various respiratory- and allergy-related outcomes in the 1946 British birth cohort. We also test for gene–gene interactions with functional simple nucleotide polymorphism (SNP) within the genes *IL13*, *IL1B*, *TNFA* and *IL1RN* which encodes the interleukin 1 receptor agonist and interacts with *IL1B* (see Table S1 in the supporting information available online), and *ERBB1* with respect to disease outcome.

## Methods

### Study participants

The MRC National Survey of Health and Development is a socially stratified sample of 5362 of all British births during 1 week of March 1946. The data collections from which we have collated information were carried out at age 43(1989) and 53 years (1999) when research nurses visited study members in their own homes and asked a series of health and lifestyle questions. At age 53, 2989 of the cohort members were interviewed. Contact was not attempted for the 1979 individuals who had previously refused to take part, were living abroad, were untraced since the previous contact at 43 years or had already died. The responding sample at age 53 is in most respects representative of the national population of a similar age^27^ and considered to be representative of a European population since the study began before mass immigration into the United Kingdom. Blood and buccal samples were collected from consenting participants at age 53 (ethical approval reference MREC no. 98/2/121).

### Outcome variables

Table 1 shows a description of all outcome variables and measures. The outcome variables indicating whether individuals had ever had asthma or hay fever were as described previously.^28^ Forced expiratory volume in 1 s and forced vital capacity, were recorded at each visit using a Micromedical turbine electric spirometer (Cardinal Health UK 232 Ltd, Basingstoke, UK).

**Table 1:** Outcome variables used; questions asked and measured in 1989 or 1999

Questions asked	Responses	Recoded variable as analysed in this study
Have you ever had asthma (1946–1989)?	No	No or yes (once/recurring) combined with question from 1999 below
	Yes, once	
	Yes, recurring	
In the last 10 years (1989–1999), did you have asthma?	No	**Ever asthma to 1999**
	Yes	
Have you ever had hay fever (1946–1989)	No	No or yes (once/ recurring) combined with question from 1999 below
	Yes, once	
	Yes, recurring	
In the last ten years (1989–1999), did you have hay fever?	No	**Ever hay fever to 1999**
	Yes	
Have you ever had bronchitis (1946–1989)?	No	**Ever bronchitis to 1989**
	Yes, once	
	Yes, recurring	
During the past 3 years (1996–1999), did you have any chest illness, such as bronchitis or pneumonia, which kept you off work or indoors for a week or more?	Yes	**Bronchitis 96–99**
	No	
Does your chest ever sound wheezy or whistling? Do you get this most days (or nights)? (Asked in 1989)	No, or not most days or nights	**Wheeze 89**
	Yes, most days or nights	
Does your chest ever sound wheezy or whistling? Do you get this most days (or nights)? (Asked in 1999)	No, or not most days or nights	**Wheeze 99**
	Yes, most days or nights	
Has this baby ever had a lower tract respiratory infection (i.e. bronchitis, bronchopneumonia or pneumonia)? (asked of mother when cohort member 24 months)	Yes	**LRTI**
	No	
Max FEV_1_ reading in 1989†	See methods	**FEV89**
Max FEV_1_ reading in 1999†	See methods	**FEV99**
Difference in Max FEV_1_ over 10 years (1989–1999).†		**DELTA**
FEV_1_/FVC ratio		**FEV_1_/FVC89 and 99**

† Three measures were recorded at age 43 and two at age 53. Only participants where at least two measures were taken, and deemed satisfactory, were included. The maximum readings were selected for analysis. Variables used shown in bold.

FEV_1_, forced expiratory volume in 1 s; FVC, forced vital capacity; LRTI, lower respiratory tract infection.

### Confounders

Potential confounders were chosen because they were previously reported to be significantly associated with one or more of the outcome variables, or considered to be of direct biological relevance.^29^–^30^ These were smoking status, childhood social class, own social class, gender and region of birth, as well as height for lung function measurements (see Table 2 for demographic details of the key outcome and confounder variables).

**Table 2:** Demographic details and frequencies of outcome variables in the test population

	All *n* (%)	Men *n* (%)	Women *n* (%)
Gender			
Male	1441 (49.4)	NA	NA
Female	1447 (50.6)	NA	NA
Own social class			
Non manual	1756 (68.1)	824 (63.1)	932 (73.3)
Manual	821 (31.9)	481 (36.9)	340 (26.7)
Father's social class			
Non manual	1139 (42.5)	558 (42.0)	581 (43.0)
Manual	1539 (57.5)	770 (43.0)	769 (57.0)
Region			
Scotland	320 (11.0)	160 (11.1)	160 (10.8)
Northern England	721 (24.7)	388 (26.9)	333 (22.6)
Central England	1187 (40.7)	572 (39.7)	615 (41.6)
London/Southeast	690 (23.7)	321 (22.3)	369 (25.0)
Ever smoking 1989			
No	813 (29.7)	347 (25.9)	466 (33.7)
Yes	1924 (70.3)	993 (74.1)	931 (66.6)
Ever asthma 1999			
No	2464 (89.9)	1210 (90.2)	1254 (89.6)
Yes	277 (10.1)	132 (9.8)	145 (10.4)
Ever hay fever 1999			
No	2081 (76.1)	1013 (75.6)	1068 (76.5)
Yes	655 (23.9)	327 (24.4)	328 (23.5)
Ever bronchitis 1989			
No	2180 (79.6)	1082 (80.7)	1098 (78.5)
Yes	560 (20.4)	259 (19.3)	301 (21.5)
Wheeze 1989			
No	2493 (91.3)	1189 (92.4)	1304 (95.8)
Yes	155 (5.9)	98 (7.6)	57 (4.2)
Wheeze 1999			
No	2664 (91.3)	1303 (90.5)	1361 (92.2)
Yes	253 (8.7)	137 (9.5)	116 (7.9)
LRTI			
No	2038 (75.3)	995 (74.3)	1043 (76.2)
Yes	670 (24.7)	344 (25.7)	326 (23.8)

The table sample includes all those that had data for the MUC genes and at least one of the outcomes.

LRTI, lower respiratory tract infection; NA, not applicable.

### Genotyping

Details of DNA extraction, genotyping and validation as well as choice of SNP are given in the online supporting information (Text and Tables S1, S2 in the supporting information available online).

### Data analyses

LDmax (http://www.sph.umich.edu/csg/abecasis/GOLD/docs/ldmax.html) was used to calculate pairwise measures of linkage disequilibrium. All further statistical analyses were performed using SPSS or STATA software. For each categorical outcome, contingency tables were constructed to compare the distribution of genotypes or alleles between the ‘affected’ and ‘unaffected’ groups with respect to disease variables. Multiple logistic regression models were then used to adjust the important associations for potential confounders. For these analyses, each of the SNP markers was coded by genotype (co-dominant model, with alleles grouped where necessary—see supplementary information). *MUC5AC* TR has two common length alleles and several rare ones which were considered as genotypes made up of three alleles L, (long) S (short) and R (rare). For binary (yes/no) outcomes, because the *MUC2* TR data are recorded as a continuous variable, we compared the *MUC2* TR allele size distributions (using a Mann–Whitney test) between the two groups, as done for our previously published study in which allele length was associated with asthma.^23^ Regression analysis was carried out to relate *MUC2* allele length to lung function, using the combined *MUC2* allele lengths for each individual and categorized into four gender-specific quartiles. Finally, potential interactions between each of the inflammatory loci and *MUC5AC* rs1132440 were explored and assessed using the likelihood ratio test. To display these differences in distribution in a simple manner graphically, we combined heterozygotes and homozygotes for the minor allele in each case.

## Results

### 11p15.5 mucin gene variants typed in the National Survey of Health and Development cohort

Details and allele frequencies for polymorphisms within *MUC2*, *MUC5AC* and *MUC5B* are shown in Table S2 in the supporting information available online. No significant deviation from Hardy–Weinberg equilibrium was observed for any polymorphism. The *MUC2* TR allele lengths ranged from 3.21 to 11.64 kb. As previously reported,^23^ ^26^ there was a major mode between 7 and 8 kb with a minor mode of around 4 to 4.5 kb.

### Linkage disequilibrium within the 11p15.5 MUC gene complex

As reported previously for other markers in this region,^26^ all of the *MUC5AC* and *MUC5B* markers are significantly associated with one or more of the others (see Table S3 in the supporting information available online).

Statistically significant association was also found between the *MUC2* TR allele length distribution and the *MUC5AC* TR genotypes, LL and LS being associated with shorter *MUC2* alleles, (*P* < 0.001, Kruskal-Wallis test) as found previously using family inferred haplotypes.^26^ The *MUC2* TR and *MUC5AC* rs1132440 showed a similar trend, although this was not statistically significant (*P* = 0.084). Thus a general pattern of association can be seen to extend from *MUC2* to *MUC5AC.*

### Tests of association between mucin genetic variants and respiratory variables

Each mucin genetic variable (see Table S2 in the supporting information available online) was analysed for association with each of the respiratory outcomes detailed in Table.1

*MUC5AC* rs1132440 genotype counts showed statistically significant association with hay fever, bronchitis and wheeze at 43 years (3 × 2 contingency tables chi-square *P*-values 0.001 to 0.02) and were marginally associated with asthma (*P* = 0.06, Table S4 in the supporting information available online). Curiously, for all outcomes, there was an increase in heterozygote frequency and a decrease in the rarer GG homozygote in affected individuals (Fig. S1 in the supporting information available online). The change in heterozygote frequency had the effect of causing a statistically significant deviation from Hardy–Weinberg equilibrium in both the yes and no groups for hay fever (*P* = 0.01). Allele count differences (2 × 2 contingency tables) were only statistically significant for bronchitis and wheeze (*P* = 0.026 and 0.027 respectively).

The *MUC5AC* TR genotype variable showed marginally significant association with hay fever (*P* = 0.044) but was not significant with any other outcomes. Because the *MUC5AC* TR dataset is somewhat smaller than the SNP dataset (Table S2 in the supporting information available online) because of the requirement for high-quality blood DNA for the Southern blot analysis, tests for association of *MUC5AC* rs 1132440 with all outcomes were also performed on the smaller dataset. Significance remained for bronchitis, wheeze and hay fever, suggesting that associations with *MUC5AC* rs 1132440 are stronger than those with *MUC5AC* TR. For the *MUC5B* SNP data, a significant association was observed between the exon 2 SNP (rs 2672785) and wheeze at 43 years (*P* = 0.022). There was a trend towards longer *MUC2* TR alleles in the asthma and wheeze groups, but this was not statistically significant.

The measures of lung function (forced expiratory volume in 1 s, forced vital capacity, Δ forced expiratory volume and forced expiratory volume in 1 s/ forced vital capacity), adjusted for gender and height showed just one significant association, namely heterozygotes for *MUC5B* rs2672785 showed slightly (1%) but significantly reduced forced expiratory volume in 1s/forced vital capacity in 1989 (*P* = 0.028). This remained significant after full adjustment for the other confounders.

Because *MUC5AC* rs1132440 showed both stronger association and association with more respiratory outcomes than any of the other loci, all further analyses were conducted using only this locus.

### Adjusting for confounders and identifying risk genotypes

In an adjusted model, all previously identified associations remain significant (Table 3).

**Table 3:** Association between *MUC5AC* rs1132440 genotypes and respiratory outcomes

Outcome	Genotypes (CC versus)	Before adjustment		After adjustment		*n*
		OR (95% CI)	*P*-value	OR (95% CI)	*P*-value	
Ever bronchitis 1989†	GC	1.042 (0.83–1.30)	0.717	1.033 (0.83–1.29)	0.779	2361
	**GG**	**0.689 (0.50–0.94)**	**0.019**	**0.683 (0.50–0.93)**	**0.017**	
Wheeze 1989 ‡	GC	0.938 (0.64–1.37)	0.739	0.898 (0.61–1.32)	0.582	2285
	**GG**	**0.382 (0.20–0.74)**	**0.005**	**0.372 (0.19–0.73)**	**0.004**	
Ever asthma 1999	GC	0.975 (0.73–1.31)	0.865	0.979 (0.73–1.31)	0.887	2362
	**GG**	**0.614 (0.40–0.94)**	**0.025**	**0.615 (0.40–0.94)**	**0.026**	
Ever hay fever 1999	**GC**	1.220 (0.99–1.51)	0.068	**1.242 (1.00–1.54)**	**0.049**	2357
	GG	0.930 (0.70–1.23)	0.614	0.949 (0.71–1.26)	0.721	

Outcomes are described in Table 1. Logistic regression OR and *P*-values are shown both before and after adjusting for the possible confounders; smoking status, region of birth, father's social class, own social class, gender. Significant associations are shown in bold and OR 95% confidence intervals are in parentheses.

† Note that this variable does not exist for 1999.

‡ Wheeze most days and nights; was not statistically significant in 1999.

CI, confidence interval; OR, odds ratio.

The rare homozygote genotypes of rs1132440 appeared to confer *protection* against bronchitis (odds ratio (95% confidence interval) = 0.689 (0.50–0.94)), wheeze (0.382 (0.20–0.74)) and asthma (odds ratio = 0.614 (0.40–0.94)) as the odds ratios are significantly less than 1 (*P*-values ≤ 0.025 in all cases) (Table 3). For hay fever, heterozygosity appeared to confer risk (adjusted odds ratio (95% confidence interval) = 1.24 (1.00–1.54); *P* = 0.049).

### The inflammatory markers

Details of the inflammatory response markers tested are given in Table S1.

Significantly different allelic distributions between the affected and unaffected groups were found for: the *IL13* promoter SNP (rs1800925) in asthma (*P* = 0.038); the *IL13*exonic SNP (rs20541) in asthma (*P* = 0.0007) *; the *ERBB1* SNP (rs2227983) in bronchitis (*P* = 0.007). For both *IL13* SNPs, the rare allele confers risk and is overrepresented in the asthmatic affected group. In contrast, the rare *ERBB1* rs2227983 allele is significantly underrepresented in the affected bronchitis group. Logistic regression analysis showed significant associations between genotype and outcome in each of these cases, which remained significant after adjustment for the potential confounders and the association between *IL1B* and asthma became significant (Table 4).

**Table 4:** Association between inflammatory mediator genotypes and respiratory outcomes

	*ERBB1* (L and S)^*^		*ERBB1* (rs2227983)		*IL13* (rs1800925)		*IL13* (rs20541)		*IL1B* (rs16944)		*IL1RN* (TR) 2 and X		*TNF* (rs1800629)	
Variable	1	2	1	2	1	2	1	2	1	2	1	2	1	2
Ever bronchitis 1989	0.96 (0.75–1.23)	1.01 (0.76–1.34)	*0.90 (0.72–1.11)*	**0.53 (0.32–0.88)**	1.22 (0.98–1.52)	1.02 (0.57–1.82)	1.03 (0.82–1.29)	1.03 (0.55–1.91)	0.93 (0.75–1.15)	1.02 (0.72–1.44)	1.08 (0.87–1.33)	0.93 (0.64–1.37)	0.92 (0.73–1.14)	0.99 (0.57–1.72)
Wheeze 1989	1.20 (0.77–1.87)	1.03 (0.60–1.77)	*0.69 (0.46–1.02)*	1.20 (0.62–2.34)	0.82 (0.54–1.25)	0.85 (0.30–2.41)	0.65 (0.41–1.02)	0.65 (0.20–2.12)	1.11 (0.76–1.64)	1.32 (0.74–2.38)	0.99 (0.67–1.47)	1.75 (0.99–3.08)	0.76 (0.50–1.16)	1.81 (0.83–3.92)
Ever asthma 1999	1.04 (0.75–1.45)	1.01 (0.69–1.49)	1.13 (0.92–1.38)	1.13 (0.76–1.66)	**1.45 (1.08–1.93)**	1.02 (0.46–2.26)	**1.47 (1.11–1.96)**	1.46 (0.68–3.14)	1.20 (0.90–1.60)	**1.54 (1.00–2.37)**	1.22 (0.92–1.63)	1.54 (0.98–2.41)	0.87 (0.64–1.17)	1.39 (0.74–2.63)
Ever hay fever 1999	1.10 (0.87–1.39)	1.02 (0.78–1.34)	0.90 (0.67–1.19)	1.15 (0.68–1.95)	**1.24 (1.00–1.53)**	0.96 (0.55–1.67)	1.13 (0.91–1.39)	1.22 (0.69–2.17)	1.12 (0.92–1.37)	0.98 (0.70–1.38)	1.04 (0.85–1.27)	1.14 (0.81–1.61)	0.84 (0.68–1.04)	1.19 (0.73–1.94)

Logistic regression odds ratios (OR) after adjusting for the confounders listed in Table 3:1 is heterozygote and 2 is homozygous for the rarer or risk allele. Significant associations are shown in bold and OR 95% confidence intervals are in parentheses. *ERBB1* microsatellite and *IL1RN* VNTR are multiallelic so to simplify analysis; the allelic data were binned into two appropriate categories, defined by reviewing the literature for allelic functional relevance. For the *ERBB1*/*EGFR* microsatellite, repeat numbers were defined as either short (S) or long (L). S being 8–18 repeats of 20 or greater denoted as L_§_. The *IL1RN* tandem repeat lengths were categorized as 2 or X; 2 referring to the IL1RN*2 allele (previously described risk allele) and X includes all other alleles (IL1RN*1, 3, 4 and 5). N values range from 2194–2361.

### Tests for gene–gene interactions

Significant interactions with respect to bronchitis were identified between *MUC5AC* rs1132440 and *ERBB1* rs2227983 (*P* = 0.019), *IL1RN* VNTR (*P* = 0.009) and *TNFA* rs1800629 (*P* = 0.046). The *ERBB1* and *IL1RN* interactions are illustrated graphically in Figure S2 in the supporting information available online. The association of *MUC5AC* with bronchitis is only significant in individuals who lack the IL1RN*2 (risk) allele (Fig. S2A in the supporting information available online) and only in individuals homozygous for the *ERBB1* common rs2227983(R) allele—that is non-carriers of the rarer K allele (Fig. S2B in the supporting information available online).

## Discussion

Abnormal expression of mucinsis a central feature of airway pathology. Here we report significant associations between a *MUC5AC* SNP rs1132440 and occurrence of asthma, wheeze, hay fever and bronchitis. Although this particular *SNP* did not show significant association with measures of respiratory function, there was a statistically significant association of forced expiratory volume in 1 s/forced vital capacity with heterozygosity for a SNP within *MUC5B* which itself is significantly associated with rs1132440. A novel association between bronchitis and an *ERBB1/EGFR* SNP rs2227983 was also identified and possible gene–gene interactions between *MUC5AC* and *ERBB1* and *IL1RN* are described.

Variations in the TR regions of the mucin genes that can potentially influence mucus rheology and mucin glycosylation were initially thought to be strong candidates for influencing inflammatory disease susceptibility. The strength of association seen here between the respiratory outcomes and *MUC5AC* rs1132440 was not however reflected by the *MUC5AC* TR data, although with hindsight, the use of alternative methods to improve resolution of individual TR alleles may have been more informative.^31^

The deviations from Hardy–Weinberg equilibrium due to an increased number of heterozygotes in the affected groups suggested that risk may be attributable to more than a simple nucleotide polymorphism and that the region may be subject to copy number variation, with the risk allele being a duplicated region. Efforts to demonstrate this, using the copy number variation detection technique multiplex ligation-dependent probe amplification^32^ or examining allelic imbalance in heterozygotes were unsuccessful, suggesting that a common duplication was not involved, so the explanation remains elusive.

The evidence of interactions between *MUC5AC* and various functional variants of genes will need replication, but the newly described interaction between *ERBB1* and *MUC5AC* is of particular interest. The *MUC5AC* association appears to be dependent upon the presence of two copies of the ancestral *ERBB1R* allele, which leads to increased signalling in response to ligand binding^33^ and consequently causes enhanced levels of inflammation, higher risk of bronchitis and presumably higher *MUC5AC* expression.

In this study, we have not corrected for multiple testing because the initial tests for association were driven by an *a priori* hypothesis and were between a series of non-independent *MUC* gene markers (associated because of their close proximity in a gene cluster) and non-independent outcome variables. The inflammatory markers were ones for which there was prior evidence of association with respiratory disease and/or there was evidence of function for the SNP themselves, but the evidence for interactions must be considered preliminary, because the three interactions for bronchitis that were significant at the 5% level were detected in a test involving seven loci and four (albeit non-independent) outcomes (28 tests).

In the main analysis, a total of six *MUC* gene markers were tested with seven categorical and five respiratory function outcomes, making a total of 72 initial/unadjusted tests for which six were significant at the 5% level. Thus a false positive association of *MUC5AC* with respiratory outcomes certainly cannot be excluded, but it is noteworthy that this gene region is now repeatedly showing association with respiratory disease. Statistically significant associations have been recently noted between several SNP in the region of *MUC5AC* and *MUC5B* and the respiratory diseases familial interstitial pneumonia and idiopathic pulmonary fibrosis.^8^ These authors claim that a single SNP about 3 kb upstream from the start of transcription of *MUC5B* is causal, because it is associated with increased expression and the rarer allele is four times more frequent in patients than controls.

A recent study has also shown an association between *MUC5AC* and cystic fibrosis respiratory disease severity,^31^ where a specific TR region allele shows strongest association.

An asthma susceptibility locus has also been mapped to 11p15 in a genome-wide linkage study of Caucasian families.^34^ While this result has not been replicated in any asthma genome-wide association studies, methods used to analyse genome-wide association studies data are extremely conservative and therefore type II errors (false negative) are likely to be extensive.

Here we have shown the strongest associations with a synonymous SNP rs1132440 in the *MUC5AC* C-terminal region which is unlikely to alter function of the protein, although the G allele is predicted using the bioinformatic software known as ESEfinder to create two exonic splicing enhancer sites (http://fastsnp.ibms.sinica.edu.tw/pages/input_CandidateGeneSearch.jsp). It seems more likely from the preliminary evidence of interaction with genes that play a role in *MUC5AC* expression that the true functional variant is within a regulatory region and affects *MUC5AC* expression. This effect is probably distinct from that observed for familial interstitial pneumonia and idiopathic pulmonary fibrosis, which is suggested to involve *MUC5B*, unless the causal SNP also affects *MUC5AC* expression, or that the inflammatory mediators also affect *MUC5B* expression. The causal locus might more likely be the same as that recently found for cystic fibrosis severity^31^, where a particular TR allele shows the best association. It is of interest that using the publically available European ‘CEPH’ data and software called ‘SNAP' (http://www.broadinstitute.org/mpg/snap/ldsearch.php), it can be seen that rs28514396, which is the best SNP for defining the risk haplotype for cystic fibrosis severity^31^, is in strong linkage disequilibrium with rs1132440 (r2 0.93; D' of 0.963). The fact that the *MUC5AC* sequence is still incomplete in the ‘complete’ human genome sequence, and the complexity of the TR region means that it may be some time before the full story can be elucidated, but there seems little doubt that variation in these genes plays a role in respiratory disease susceptibility.

## Footnotes

* The associations between both *IL13* SNP and the asthma outcome on this dataset have been reported previously.^28^

## Abbreviations

TR: tandem repeat

## Supporting Information

**Figure S1** Bar charts showing as percentages the *MUC5AC* rs1132440 genotype frequency distribution in the affected and unaffected groups for outcomes bronchitis 1989, wheeze most days and nights 1989, ever asthma (1999) and ever hay fever (1999). See Table S1 for n values. Note that in each case the heterozygotes show elevated frequencies in the yes group while the rarer homozygotes show lower frequency. **Figure S2** Bar charts showing the distributions as percentages of the *MUC5AC* rs 1132440 genotypes in carriers and non-carriers of minor/risk alleles of *ERBB1* and *IL1RN* with respect to Ever Bronchitis. A *IL1RN* ^*^2 a) carriers of the *IL1RN* risk allele (22, 23, 24, 25, 26) (NS).b) non-carriers of the *IL1RN* risk allele (34, 44, 45, 46, 55) *P* = 0.004. B *ERBB1* rs2227983. (a) carriers of the rare *ERBB1* K allele (AA and AG) (NS). (b) non-carriers (GG, and homozygous for the ancestral allele R) *P* < 0.001. *P*-value from Pearson chi-square; NS, not significant. **Table S1** Details of variants within the genes encoding the inflammatory mediators and functional evidence for these. Note that the marker names refer to physical positions within the gene or its regulatory sequence. ‡ rs2227983 was previously listed as rs11543848 (NBCI dbSNP). Minor allele frequencies (MAF) range from 0.17 to 0.49 and genotype distributions were in accordance with Hardy-Weinberg equilibrium, with the exception of *IL1B* rs16944 (*P* = 0.01). **Table S2** *MUC5AC* and *MUC5B* polymorphisms typed on the 1946 cohort. Note that for simplicity the *MUC5AC* TR alleles have been categorized into a tri-allelic model where r refers to all rare alleles. **Table S3** Pairwise linkage disequilibrium (LD) measures for *MUC5AC* and *MUC5B* markers. (a) Significance of association is shown as chi-square *P*-values. (b) D′ measure of LD. Loci are shown in chromosomal order from *MUC5AC* TR through to rs2075859 in exon 9 of *MUC5B* (see Table S1) Note that all adjacent SNP are highly associated with each other. Although there is breakdown of LD in between exons 2 and 9 of *MUC5B*, with no significant association between rs2672785 and rs2075859, LD is still detectable across the two *MUC5* genes since *MUC5AC* rs1132440 and *MUC5ACTR* are each significantly associated with one of the *MUC5B* SNP even though the D' values are small. Significant values are shown in bold. **Table S4** Chi-square *P*-values from contingency tables of *MUC5AC and MUC5B* genotypes and the categorical respiratory outcomes; Mann-Whitney *P*- value for *MUCTR.* N values given are for MUC5AC rs1132440; those for other loci, in particular *MUC2* TR and *MUC5AC* TR are a little lower (see manuscript text). y/n signifies yes or no as indicated on Table 1. Similar empirical *P*-values were obtained by permutation analysis.
