# Supplementary material for: MUC5AC and inflammatory mediators associated with respiratory outcomes in the British 1946 birth cohort
Source: Respirology. 2013 Jul 25;18(6):1003–10. doi: 10.1111/resp.12092 (PMC3784974; doi:10.1111/resp.12092)

Table S1 Details of variants within the genes encoding the inflammatory mediators and functional evidence for these. Note that the marker names refer to physical positions within the gene or its regulatory sequence. _‡_ rs2227983 was previously listed as rs11543848 (NBCI dbSNP). Minor allele frequencies (MAF) range from 0.17 to 0.49 and genotype distributions were in accordance with Hardy Weinberg Equilibrium, with the exception of *IL1B* rs16944 (p = 0.01).

| **Gene** | **Polymorphism** | **Location** | **Marker Nomenclature** | **Alleles** | **MAF** | **Literature** |
| --- | --- | --- | --- | --- | --- | --- |
| ***ERBB1/EGFR*** | **Microsatellite** | Intron 1 | none | L>S _§_ | 0.49 (S) | Transcriptional activity of *ERRB1* declines with increasing numbers of microsatellite repeats (^7^). The shorter alleles have been significantly associated with asthma (^8^). Role in relation to *MUC5AC* (^9-13^) |
| ***ERBB1/ EGFR*** | **rs2227983_‡_** | Exon 13 | R497K, R521K | G>A | 0.26 (A) | The presence of the lysine variant weakens the EGFR growth response to its ligands (^14^). Role in relation to *MUC5AC* (^9-13^) |
| ***IL13*** | **rs1800925** | Promoter | C-1024T, C-1111T  C-1112T, C-1055T | C>T | 0.18 (T) | Experiments on T cells from individuals homozygous for the rarer allele showed that this genotype confers a more robust expression pattern Genetically associated with asthma. (^5;15;16^) (^17^) Role in relation to *MUC5AC(^18;19^)* |
| ***IL13*** | **rs20541** | Exon 4 | R110Q, R130Q | G>A | 0.17 (A) | Enhanced binding of IL13 to its receptor when the Glutamine allele (Q) is present. Genetically associated with inflammatory disease. Q allele is also associated with higher IgE levels (^5;20-25^) Role in relation to *MUC5AC(^18;19^)* |
| ***IL1B*** | **rs16944** | Promoter | G-511A | G>A | 0.34 (A) | Confers higher levels of IL1Ra when in combination with a particular *IL1RN* VNTR allele (^26^). Haplotypes of this variant and the *IL1RN* VNTR were shown to be significantly associated with decline of lung function in smokers (^27^). Role in relation to *MUC5AC(^28;29^)* |
| ***IL1RN/ IL1Ra*** | **VNTR** | Intron 2 | none | X>2_‡_ | 0.29 (2) | Significantly associated with asthma and with IL1Ra serum levels (^26;27;30-34^) Role in relation to *MUC5AC see above* |
| ***TNFA*** | **rs1800629** | Promoter | G-308A, G-488A | G>A | 0.19 (A) | The A variant is associated with higher level of expression than the G and significantly associated with asthma in many independent studies (^35-44^) Role in relation to *MUC5AC(^45^)* |

**Table S2** ***MUC5AC* and *MUC5B* polymorphisms typed on the 1946 cohort.** Note that for simplicity the *MUC5AC* TR alleles have been categorised into a tri-allelic model where r refers to all rare alleles.

| **Gene** | **SNP location** | **SNP ID** | **allele frequency** | **Total** |
| --- | --- | --- | --- | --- |
| ***MUC5AC*** | VNTR | 5ACTR | 0.77 (S)  0.22 (L)  0.01 (r) | 2673 |
| ***MUC5AC*** | exon 19 (3′ region) | rs1132440 | 0.42 (G) | 2910 |
| ***MUC5B*** | exon 2 (5′ region) | rs2672785 | 0.20 (G) | 2800 |
| ***MUC5B*** | exon 3 (5′ region) | rs2075853 | 0.07 (T) | 2801 |
| ***MUC5B*** | exon 9 (5′ region) | rs2075859 | 0.38 (T) | 2797 |

**Table S3 Pairwise linkage disequilibrium (LD) measures for *MUC5AC* and *MUC5B* markers.** a) **Significance of association is shown as chi squared P-values. b) D′**  **measure of LD. Loci are shown in chromosomal order from *MUC5AC TR*  through to rs2075859 in exon 9 of *MUC5B (see Supplementary Table 1)*** Note that all adjacent SNPs are highly associated with each other. Although there is breakdown of LD in between exons 2 and 9 of *MUC5B*, with no significant association between rs2672785 and rs2075859, LD is still detectable across the two *MUC5* genes since *MUC5AC* rs1132440 and *MUC5ACTR* are each significantly associated with one of the *MUC5B* SNPs even though the D′ values are small. Significant values are shown in bold.

| a) |  |  |  |  |  |
| --- | --- | --- | --- | --- | --- |
| **5ACTR** |  |  |  |  |  |
| **rs1132440** | **0.000** |  |  |  |  |
| **rs2672785** | 0.751 | **0.000** |  |  |  |
| **rs2075853** | **0.047** | 0.264 | **0.000** |  |  |
| **rs2075859** | 0.803 | **0.009** | 0.968 | **0.000** |  |
|  | **5ACTR** | **rs1132440** | **rs2672785** | **rs2075853** | **rs2075859** |
|  | ***MUC5AC*** | | ***MUC5B*** | | |

| b) |  |  |  |  |  |
| --- | --- | --- | --- | --- | --- |
| **5ACTR** |  |  |  |  |  |
| **rs1132440** | **0.941** |  |  |  |  |
| **rs2672785** | 0.014 | **0.198** |  |  |  |
| **rs2075853** | **0.065** | 0.062 | **0.910** |  |  |
| **rs2075859** | 0.016 | **0.071** | 0.001 | **0.958** |  |
|  | 5ACTR | rs1132440 | rs2672785 | rs2075853 | rs2075859 |
|  | ***MUC5AC*** | | ***MUC5B*** | | |

Table S4 Chi square p-values from contingency tables of *MUC5AC and MUC5B* genotypes and the categorical respiratory outcomes; Mann Whitney p-value for *MUCTR.* N values given are for MUC5AC rs1132440; those for other loci, in particular MUC2TR and MUC5ACTR are a little lower (see manuscript text). y/n signifies yes or no as indicated on Table 1. Similar empirical p values were obtained by permutation analysis.

| **Outcome** | ***MUC2***  ***TR*** | **N***  **y/n** | ***MUC5AC* rs1132440** | ***MUC5AC* TR** | ***MUC5B* rs2672785** | ***MUC5B* rs2075853** | ***MUC5B rs2075859*** |
| --- | --- | --- | --- | --- | --- | --- | --- |
|  | p value |  | p value | p value | p value | p value | p value |
| **Ever Asthma to 1999** | 0.394 | 277/2457 | 0.061 | 0.583 | 0.666 | 0.252 | 0.882 |
| **Ever Hayfever to 1999** | 0.589 | 655/2074 | **0.003** | **0.044** | 0.689 | 0.167 | 0.251 |
| **Wheeze 89** | 0.266 | 155/2486 | **0.019** | 0.216 | **0.022** | 0.317 | 0.398 |
| **Wheeze 99** | 0.658 | 253/2656 | 0.178 | 0.321 | 0.421 | 0.130 | 0.233 |
| **Ever Bronchitis to 1989** | 0.239 | 517/1999 | **0.016** | 0.377 | 0.188 | 0.958 | 0.887 |
| **Bronchitis 96-99** | 0.222 | 298/2611 | 0.111 | 0.909 | 0.369 | 0.306 | 0.973 |
| **LRTI** | 0.550 | 667/2033 | 0.859 | 0.555 | 0.670 | 0.220 | 0.275 |

Figure legends

**Figure S1 Bar charts showing as percentages the *MUC5AC* rs1132440 genotype frequency distribution in the affected and unaffected groups** for outcomes bronchitis 1989 , wheeze most days and nights 1989, ever asthma (1999) and ever hay fever (1999). See Table S1 for n values. Note that in each case the heterozygotes show elevated frequencies in the yes group while the rarer homozygotes show lower frequency.


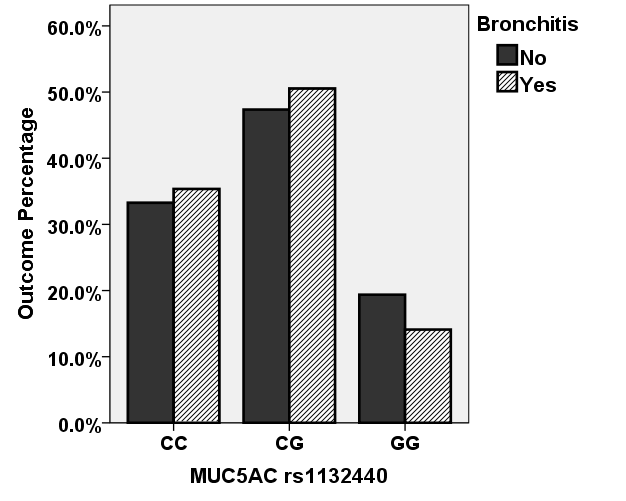

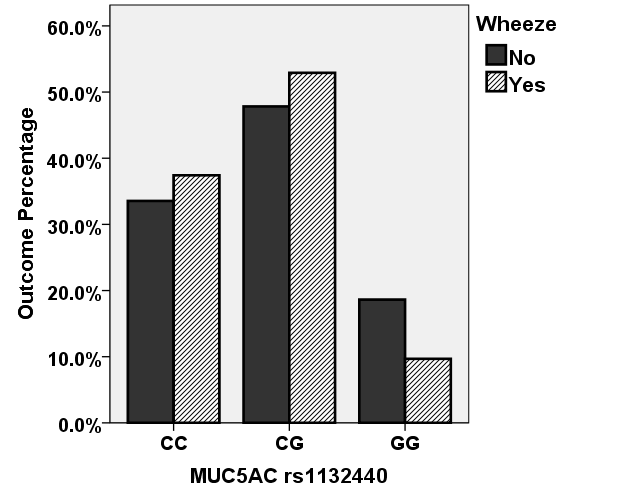


**Figure 2** **Bar charts showing the distributions as percentages of the MUC5AC rs 1132440 genotypes in carriers and non carriers of minor/risk alleles of *ERBB1* and *IL1RN* with respect to Ever Bronchitis.**


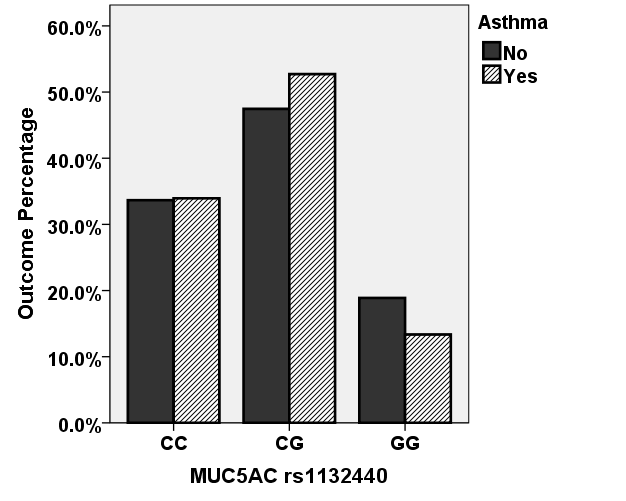

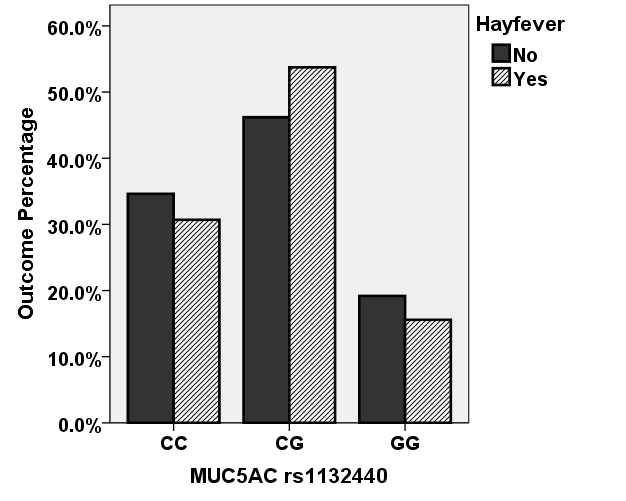


A *IL1RN* *2 a) carriers of the *IL1RN* risk allele (22, 23, 24, 25, 26) (NS).b) non-carriers of the *IL1RN* risk allele (34, 44, 45, 46, 55) P=0.004.

B *ERBB1* rs2227983. a) carriers of the rare *ERBB1* K allele (AA and AG) (NS) . b) non-carriers (GG, and homozygous for the ancestral allele R) P<0.001 .

P value from Pearson chi square, NS - not significant.

A)


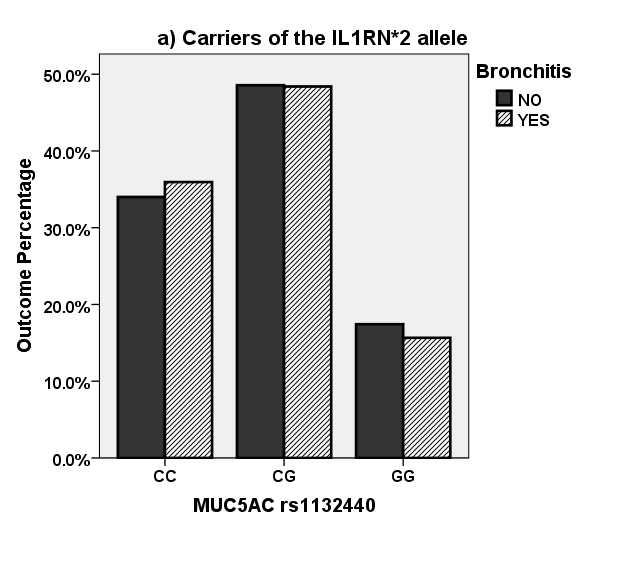

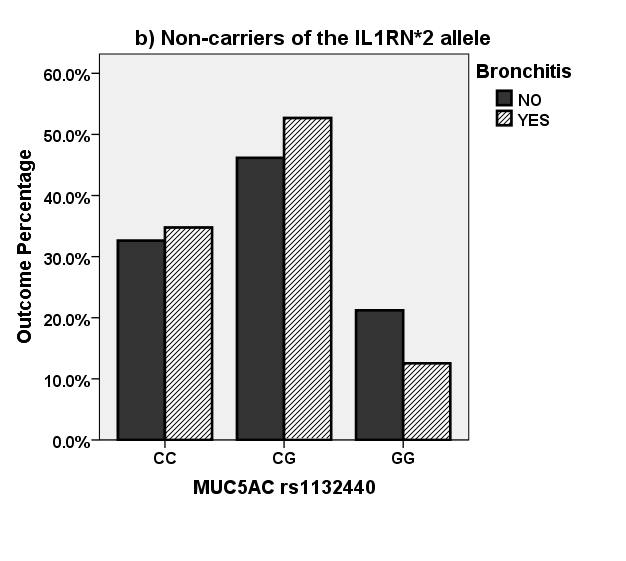


B)


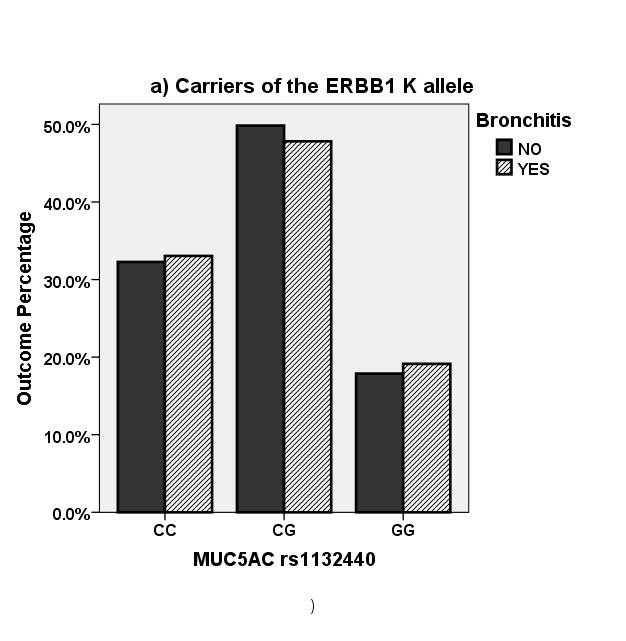

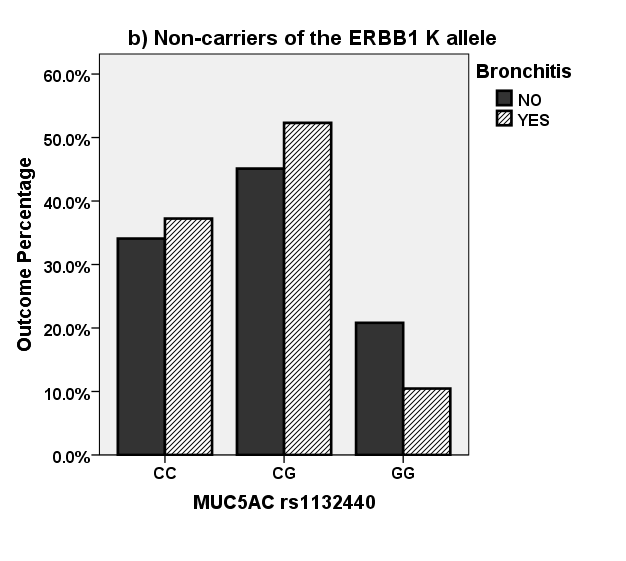

Supplement: Supplementary file 1 — Figure S1 Bar charts showing as percentages the MUC5AC rs1132440 genotype frequency distribution in the affected and unaffected groups for outcomes bronchitis 1989, wheeze most days and nights 1989, ever asthma (1999) and ever hay fever (1999). See Table S1 for n values. Note that in each case the heterozygotes show elevated frequencies in the yes group while the rarer homozygotes show lower frequency. Figure S2 Bar charts showing the distributions as percentages of the MUC5AC rs 1132440 genotypes in carriers and non-carriers of minor/risk alleles of ERBB1 and IL1RN with respect to Ever Bronchitis. A IL1RN *2 a) carriers of the IL1RN risk allele (22, 23, 24, 25, 26) (NS).b) non-carriers of the IL1RN risk allele (34, 44, 45, 46, 55) P = 0.004. B ERBB1 rs2227983. (a) carriers of the rare ERBB1 K allele (AA and AG) (NS). (b) non-carriers (GG, and homozygous for the ancestral allele R) P < 0.001. P-value from Pearson chi-square; NS, not significant. Table S1 Details of variants within the genes encoding the inflammatory mediators and functional evidence for these. Note that the marker names refer to physical positions within the gene or its regulatory sequence. ‡ rs2227983 was previously listed as rs11543848 (NBCI dbSNP). Minor allele frequencies (MAF) range from 0.17 to 0.49 and genotype distributions were in accordance with Hardy-Weinberg equilibrium, with the exception of IL1B rs16944 (P = 0.01). Table S2 MUC5AC and MUC5B polymorphisms typed on the 1946 cohort. Note that for simplicity the MUC5AC TR alleles have been categorized into a tri-allelic model where r refers to all rare alleles. Table S3 Pairwise linkage disequilibrium (LD) measures for MUC5AC and MUC5B markers. (a) Significance of association is shown as chi-square P-values. (b) D′ measure of LD. Loci are shown in chromosomal order from MUC5AC TR through to rs2075859 in exon 9 of MUC5B (see Table S1) Note that all adjacent SNP are highly associated with each other. Although there is breakdown of LD in betw [file resp0018-1003-sd1.docx]
